# Study on essential oils from four species of Zhishi with gas chromatography–mass spectrometry

**DOI:** 10.1186/1752-153X-8-22

**Published:** 2014-04-03

**Authors:** Yuanyan Liu, Zhenli Liu, Chun Wang, Qinglin Zha, Cheng Lu, Zhiqian Song, Zhangchi Ning, Siyu Zhao, Xinmiao Lu, Aiping Lu

**Affiliations:** 1 School of Chinese Materia Medica, Beijing University of Chinese Medicine, Beijing Municipal Key Laboratory for Basic Research of Chinese Medicine, Beijing 100102, China; 2 Institution of Basic Theory, China Academy of Chinese Medical Sciences, Beijing 100700, China; 3 Institute of Basic Research in Clinical Medicine, China Academy of Chinese Medical Sciences, Beijing 100700, China; 4 School of Chinese Medicine, Hong Kong Baptist University, Hongkong, China

**Keywords:** *Citrus* fruits, Essential oils, GC-MS, PCA, Zhishi

## Abstract

**Background:**

*Citrus* fruits are widely used as food and or for medicinal purposes, and they contain a host of active substances that contribute to health. The immature fruits of *Citrus sinensis* Osbeck and its cultivars (*CS*), *C. junos* Sieb. ex Tanaka (*CJ*), *C. aurantium* L. and its cultivars (*CA*) and *Poncirus trifoliate* Raf. (*PT*) are the most commonly used medicinal herbs in Traditional Chinese Medicine, called Zhishi. And their mature fruits can be used as food.

**Results:**

In this study, the essential oils of four different Zhishi species were extracted by steam distillation and detected using gas chromatography- mass spectrometry (GC-MS). A total of 39 volatiles from the four species were tentatively identified. The limonene was the most abundant amongst the four species. Principal component analysis (PCA) of essential oils showed a clear separation of volatiles among *CS*, *CJ* and *PT*. However, *CA* could not be separated from these three species. Additionally, the volatiles accounting for the variations among the widely separated species were characterized through their corresponding loading weight.

**Conclusion:**

Sesquiterpenes were identified as characteristic markers for *PT*. The content of some monoterpenes could be as taxonomic markers between *CS* and *CJ.* This work is of great importance for the evaluation and authentication of Zhishi samples through essential oils.

## Background

*Citrus* genus is the most important fruit tree crop in the world, with an annual production of approximately 102 million tons [1]. *Citrus* fruits are used in the food, cosmetic and pharmaceutical industry. They are consumed fresh and processed, as juices, jam, jellies, molasses, etc.. The fresh fruits are important starting materials for juice production [2]. Especially, chemical industry extracts from *Citrus* bioactive compounds like flavonoids, vitamins, dietary fiber and essential oils, etc. have beneficial effects on human health. The essential oils of *Citrus* fruits are widely used as flavoring in foods, perfumes and pharmaceutical formulations due to their functional properties like antimicrobial, antifungal, as well as analgesics, cough suppressants, and expectorants for eliminating phlegm [3-8]. *Citrus* oils extraction methods include steam distillation [9], cold pressing [10] and static headspace solid-phase microextraction [11].

The immature fruits of *CA*, *CS*, *CJ* and *PT* can be used as Zhishi, one of the primary traditional Chinese medicinal plants in China [12,13]. The herbs from different species and cultivation locations are widely used in clinical applications. Such herbs have been used to treat stuffiness, intestinal fullness sensation and distending pain, diarrhea and dysentery in addition to the retention and preservation of food [14]. However, the efficacy of these species of Zhishi for treating digestive disturbances varies due to differences in the types and quantities of the chemical substances they contain [15,16]. Our previous studies based on four flavanone compounds demonstrate that compounds from different species of Zhishi, such as *CA*, *CS* and *CJ,* can be distinguished by established differential modes of acquisition [17]. In addition to flavanones, essential oils are an important type of pharmacodynamic substance in Zhishi. Therefore, it is important to elucidate the differences between volatile substances among different species. Numerous studies [18-23] on the essential oils of Zhishi have been conducted using GC and GC-MS. However, because there is still a lack of information regarding the essential oils of different species of Zhishi, a comparative study is necessary.

Therefore, in this study, 75 authentic Zhishi samples from four species of *Citrus* fruit (*CA*, *CS, CJ* and *PT*) were collected. The essential oils were obtained by using steam distillation, and then were separated and identified by GC-MS. In total, 39 volatiles were tentatively identified and quantified, and among them were 26 monoterpenes, 8 sesquiterpenes, and 5 esters, aliphatic alcohols and aliphatic aldehydes. The details were shown in the Additional file 1. Then, a multivariate statistical analysis method was employed to elucidate the variation of volatile substances among different species of Zhishi. This is the first report on comparison study of essential oils from these four Zhishi species.

## Results and discussion

### Quantification of the volatile components

In total, 39 compounds were tentatively identified, constituting approximately 85.14-100% of the entire volatile concentration (Table 1). Each compound was identified by matching its retention characteristics and MS fragmentation patterns against standards and library spectra. The results are expressed as relative weight percentages calculated from peak areas. Volatiles were categorized into five chemical groups consisting of monoterpenes, sesquiterpenes, esters, aliphatic alcohols and aliphatic aldehydes. For all of the four *Citrus* species, the total content of essential oils primarily consisted of 26 monoterpenes, which accounted for 52.17-100% of the entire volatile concentration. The other four chemical groups – sesquiterpenes, esters, aliphatic alcohols and aliphatic aldehydes – contained in *CA*, *CS* and *CJ* had minor contributions to the entire volatile concentration accounting for only 0–2.28%. Sesquiterpenes were present in the oils analyzed in the greatest amount (3.8-34.28%) in the *PT* species compared to the other three species. This result is not surprising as sesquiterpenes hydrocarbons are important in the characteristic aroma of many kinds of *Citrus* fruits [24]. Alpha and β-pinene are chemically unstable bicyclic terpenes due to a strained four membered ring. Thus, they are found at low levels and evenly distributed in all the four species.

**Table 1:** Identifications of Zhishi volatiles and their relative total ion current peak area from four different species

**No.**	**Name**	**RI**	***CA-1 ~ CA-35***		***CJ-1 ~ CJ-15***		***CS-1 ~ CS-21***		***PT-1 ~ PT-4***	
			***Relative concentration***	**Mean**	***Relative concentration***	**Mean**	***Relative concentration***	**Mean**	***Relative concentration***	**Mean**
1	α-Thujene	943	0-1.15%	0.40%	0.35-1.71%	1.12%	0-0.09%	0.03%	\	\
2	α-Pinene	948	0.28-2.97%	1.25%	0.88-3.97%	2.61%	0.25-0.75%	0.50%	0-1.52%	0.58%
3	(+)-Sabinene	979	0.1-8.26%	1.77%	0-2.21%	0.54%	3.61-12.82%	7.72%	0.55-2.54%	1.35%
4	β-Pinene	982	0.4-17.01%	2.59%	1.64-3.96%	2.80%	0.35-1.01%	0.62%	0-3.46%	1.83%
5	β-Myrcene	992	0-2.72%	1.05%	0.55-1.55	0.94%	0-1.51%	1.14%	8.60-28.91%	18.29%
6	α-Phellandrene	1004	0-0.42%	0.05%	0.04-0.37%	0.09%	0-0.05%	0.02%	0.84-9.00%	4.74%
7	(+)-4-Carene	1015	0-0.88%	0.33%	0.54-1.23%	0.88%	0.08-0.35%	0.17%	0-0.18	0.09%
8	o-Cymene	1022	0-5.86%	0.63%	0.38-4.96%	1.51%	0-0.22%	0.03%	0-0.23%	0.11%
9	Limonene	1026	27-82.84%	63.47%	35.53-60.60%	46.47%	72.75-89.35%	82.25%	21.19-69.19%	48.14%
10	β-trans-Ocimene	1033	0-0.66%	0.06%	0-0.57%	0.06%	0-1.09%	0.15%	0-0.57%	0.15%
11	β-cis-Ocimene	1042	0-9.99%	2.21%	0-0.89%	0.38%	0-1.02%	0.31%	1.46-4.96%	2.60%
12	γ-Terpinene	1052	0.25-32.31%	12.01%	21.71-46.74%	34.16%	0.31-2.63%	0.91%	0-0.73%	0.40%
13	cis-β-Terpineol	1060	0-0.52%	0.06%	0-0.13%	0.05%	0-0.29%	0.12%	\	\
14	p-Mentha-1,4(8)-diene	1081	0.07-1.79%	0.66%	0.95-2.33%	1.63%	0-0.15%	0.08%	0-0.12%	0.05%
15	β-Linalool	1089	0-26.15%	9.10%	0.73-7,95%	3.53%	1.34-9.21%	4.13%	0.37-1.18%	0.66%
16	trans-1-methyl-4-(1-methylethyl)-2-Cyclohexen-1-ol	1112	0-1.1%	0.04%	\	\	0-0.05%	\	0-0.21%	0.07%
17	cis-1-methyl-4-(1-methylethyl)-2-Cyclohexen-1-ol	1129	0-0.07%	\	\	\	\	\	0-0.12%	0.03%
18	β-Citronellal	1141	0-0.2%	0.04%	0-0.05%	0.01%	0-0.06%	0.02%	\	\
19	(−)-Terpinen-4-ol	1164	0-3.23%	0.61%	0.21-1.02%	0.48%	0.33-2.17%	0.73%	0-2.42%	0.88%
20	α-Terpieol	1176	0-1.93%	0.60%	0.30-0.85%	0.60%	0.08-0.63%	0.30%	0-2.10%	0.75%
21	cis-Carveol	1202	0-.095%	0.03%	\	\	\	\	\	\
22	β-Citronellol	1210	0-0.48%	0.06%	0-0.04%	\	0-0.03%	\	0-0.30%	0.08%
23	β-Citral	1224	0-0.72%	0.06%	0-0.04%	\	0-0.21%	0.11%	\	\
24	trans-Geraniol	1236	0-1.16%	0.05%	\	\	0-0.20%	0.02%	\	\
25	α-Citral	1251	0-0.35%	0.04%	0-0.03%	0.01%	0-0.24%	0.12%	\	\
26	p-Cymen-2-ol	1269	0-1.29%	0.11%	0-1.23%	0.55%	\	\	\	\
	Monoterpenes (Total)		84.67-99.61%	96.88%	89.31-99.81%	98.42%	85.30-100%	99.48%	52.17-96.06%	80.80%
27	n-Octanal	1002	0-0.87%	0.33%	0-0.27%	0.08%	0-0.22%	0.09%	\	\
28	n-Decanal	1191	0-0.52%	0.06%	0-0.07%	0.01%	0-0.13%	0.02%	\	\
	Aliphatic Aldehydes (Total)		0-1.39%	0.39%	0-0.38%	0.09%	0-0.35%	0.11%	\	\
29	1-Octanol	1064	0-0.21%	0.02%	\	\	0-0.04%	\	0-0.03%	0.01%
30	α-Methyl-α-[4-methyl-3-pentenyl]oxiranemethanol	1065	0.04-1.26%	0.35%	0-0.38%	0.11%	\	\	\	\
	Aliphatic Alcohols (Total)		0.04-1.26%	0.37%	0-0.38%	0.11%	0-0.04%	\	0-0.03%	0.01%
31	Nerol acetate	1334	0-0.17%	0.03%	\	\	\	\	0-0.55%	0.14%
	Esters (Total)		0-0.17%	0.03%	\	\	\	\	0-0.55	0.14%
32	β-Elemene	1362	0-0.21%	0.01%	\	\	\	\	0-0.96%	0.27%
33	Caryophyllene	1387	\	\	\	\	\	\	0-14.7%	5.32%
34	β-Farnesene	1418	\	\	0-0.15%	0.03%	0-0.14%	0.01%	0.22-8.94%	2.80%
35	Germacrene D	1442	0-0.99%	0.26%	0-0.55%	0.15%	\	\	0.49-4.75%	1.83%
36	4-isopropylidene-1-vinyl-o-Menth-8-ene	1452	0-0.08%	0.01%	0-0.05%	0.01%	\	\	0-0.25%	0.08%
37	(Z,E)-α-Farnesene	1462	0-0.05%	\	0-0.08%	0.01%	0-0.03%	\	0-1.31%	0.33%
38	γ-Elemene	1493	0-0.19%	0.01%	\	\	\	\	0.37-10.60%	3.81%
39	α-Sinensal	1567	\	\	\	\	0.01-0.25%	0.01%	\	\
	Sesquiterpenes (Total)		0-1.32%	0.29%	0-0.75%	0.20%	0-0.71%	0.01%	3.8-34.28%	14.44%

Listed values are the percentage of the total peak area for each species. '\' Not detectable. The detailed data of the 75 samples from the four species is listed in the Additional file 1.

### GC-MS analyses of the four different species of Zhishi samples

In the 35 samples of *CA*, monoterpenes produced the highest percentage of volatiles (84.67-99.61%). Among the volatiles, the most predominant was limonene (27–82.84%, *Mean* 63.47%), followed by γ-terpinene (0.25-32.31%, *Mean* 12.01%), β-linalool (0–26.15%, *Mean* 9.10%), β-pinene (0.4-17.01%, *Mean* 2.59%), β-cis-ocimene (0–9.99%, *Mean* 2.21%) and (+)-sabinene (0.1-8.26%, *Mean* 1.77%). Meanwhile, the mean values of volatiles such as α-pinene, β-myrcene, p-mentha-1,4(8)-diene, o-cymene, (−)-terpinene-4-ol and α-terpieol were higher than 0.5%, with an average percentage of total volatile concentration of 1.25%, 1.05%, 0.66%, 0.63%, 0.61% and 0.60%, respectively. Other chemicals, such as germacrene D, α-methyl-α-[4-methyl-3-pentenyl]-oxiranemethanol and n-octanal, belonging to sesquiterpenes, aliphatic alcohols and aliphatic aldehydes chemical groups, were minor volatile components in *CA* species, averaging 0.26%, 0.35% and 0.33% of the total percentage, respectively.

For *CS* species, monoterpenes were also the highest concentration of the volatile, averaging 85.3-100% of the total percentage. Limonene was the most abundant monoterpene in this species accounting for 72.75-89.35%, with an average value at 82.25%. Additionally, (+)-sabinene and β-linalool were recorded at a relatively lower proportion, 3.61-12.82% and 1.34-9.21%, respectively. Other compounds within the monoterpenes group, such as β-myrcene, γ-terpinene, (−)-terpinene-4-ol, β and α-pinene, also occurred at a higher proportion, the mean amounts were 1.14%, 0.91%, 0.73%, 0.62% and 0.50%, respectively. Compounds of other chemical groups had a minor amount, and the mean values were lower than 0.1%.

For *CJ* species, the total amounts of monoterpene compounds ranged from 89.31-99.81%. The most two abundant essentials, limonene and γ-terpinene accounted for 35.53-60.60% (*Mean* 46.47%) and 21.71-46.74% (*Mean* 34.16), respectively. Other monoterpenes, such as β-linalool, β-pinene, α-pinene, p-mentha-1,4(8)-diene, o-cymene, α-thujene, β-myrcene, (+)-4-carene, α-terpieol, p-cymen-2-ol and (+)-sabinene, each had average amounts higher than 0.5% at 3.53%, 2.80%, 2.61%, 1.63%, 1.51%, 1.12%, 0.94%, 0.88%, 0.60%, 0.55% and 0.54%, respectively. Except for germacrene D, which had an average value at 0.15% and belongs to the sesquiterpenes, compounds of other chemical groups were less than 0.1%.

Interestingly, in the *PT* species, sesquiterpenes (3.80-34.28%) were an important chemical group of character impact volatiles because of the relatively high percentage in the overall volatile composition compared to the other three species. In addition, it serves a characteristic aroma for many types of *Citrus* fruits. Among these sesquiterpenes, the most predominant was caryophyllen (*Mean* 5.32%), followed by γ-elemene (*Mean* 3.81%), β-farnesene (*Mean* 2.80%) and germacrene D (*Mean* 1.83%). Limonene and β-myrcene were the major monoterpenes in this species as the average value accounted for up to 48.14%and 18.29%, respectively.

### Principal component analysis

The variability of essential oils in the four *Citrus* species of Zhishi shown in Table 1 was not anticipated. Principal component analysis (PCA) was employed to examine this large data set without having to assign classifications before analysis. PCA is an unsupervised clustering method that requires little prior knowledge of the data set and acts to reduce the dimensionality of multivariate data without losing important information. Par-scaled (scaled to square root of standard deviation) mathematical methods were performed to pre-treat the data set resulting from the volatiles of different *Citrus* species. The multivariate statistical analysis methodology helps to identify inherent patterns in the data in an unbiased manner and highlights the similarities and differences amongst samples. This methodology also helps identify those essential oils that are the most representative within the entire data set.

#### PCA score plot

**Figure 1:**
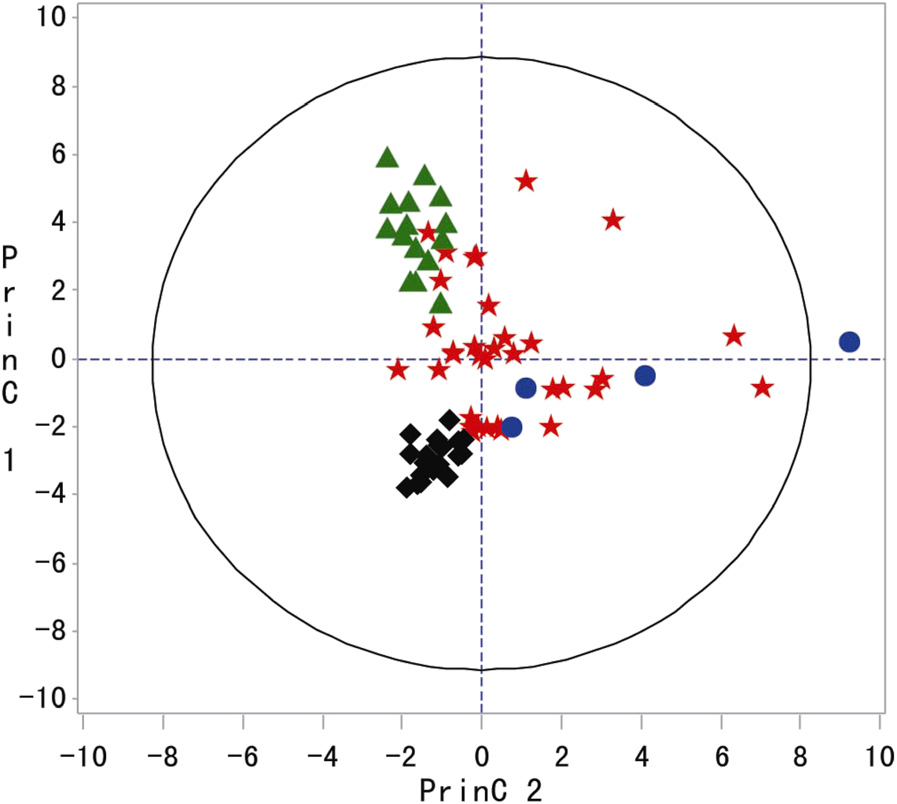
**PCA scores plot (PC1 vs. PC2) of the four species of Zhishi samples: 35 samples from *CA*, 15 samples from *CJ, *21 samples from *CS *and 4 samples from *PT*.** (*CA* = (red star) *CJ* = (green triangle), *CS* = (black diamond) and (PT = blue circle)).

As shown in Figure 1, each coordinate represents a sample, and the separation of the four different species of Zhishi samples was observed in the PCA scores plot. A two-component PCA model cumulatively accounted for 54.5% of the total variance. The *CA* species could not be separated from the other three species; however, according to our previous studies [17], *CS*, *CJ* and *CA* could be differentiated from one other by choosing flavanones as marker compounds. The high degree of clustering and minimal overlap of *CS*, *CJ* and *PT* suggest that these three species have volatile profiles that are unique to each cultivar.

**Figure 2:**
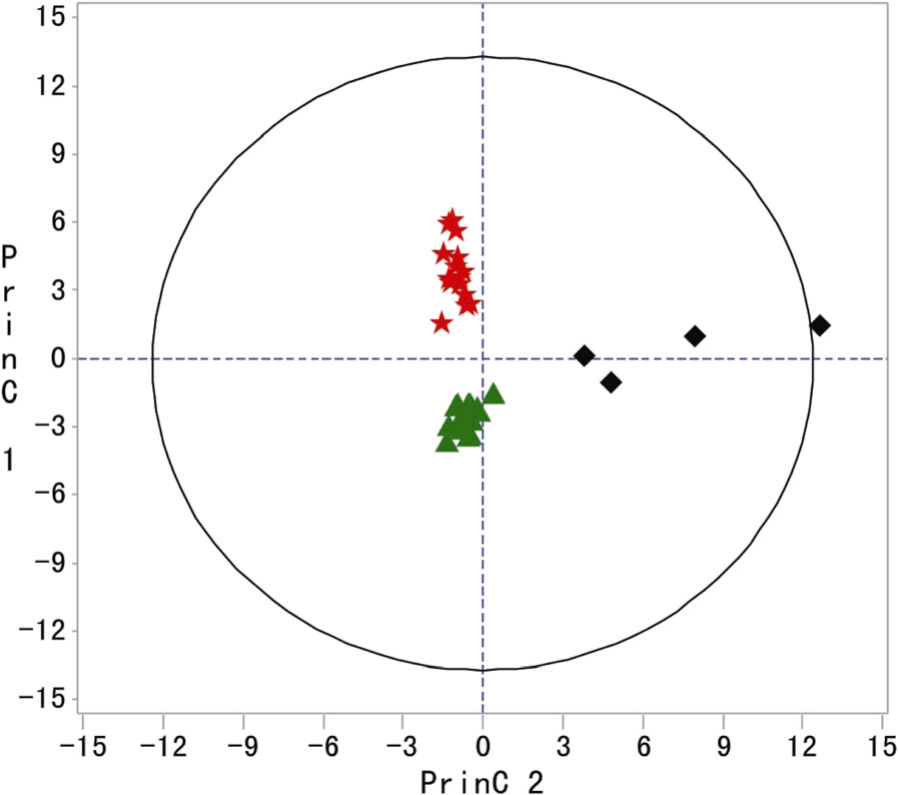
**PCA scores plot (PC1 vs. PC2) of the three species of Zhishi samples: 15 samples from *CJ*, 21 samples from *CS *and 4 samples from *PT*.** (*CJ* = (red star), *CS* = (green triangle) and *PT* = (black diamond)).

These three species, *CS, CJ, and PT*, were further analyzed by PCA. We observed three widely separated clusters in Figure 2 consisting of *CS*, *CJ* and *PT*. The *PT* samples were clearly separated from *CS and CJ* by principal component 2 (PC2), whereas the *CJ* and *CS* samples were clearly separated by principal component 1 (PC1). The results confirmed that *CS*, *CJ* and *PT* were different regarding the levels and occurrence of these essential oils. However, the volatiles in *CA* could not be distinguished from the other three species.

#### PCA loading plot

**Figure 3:**
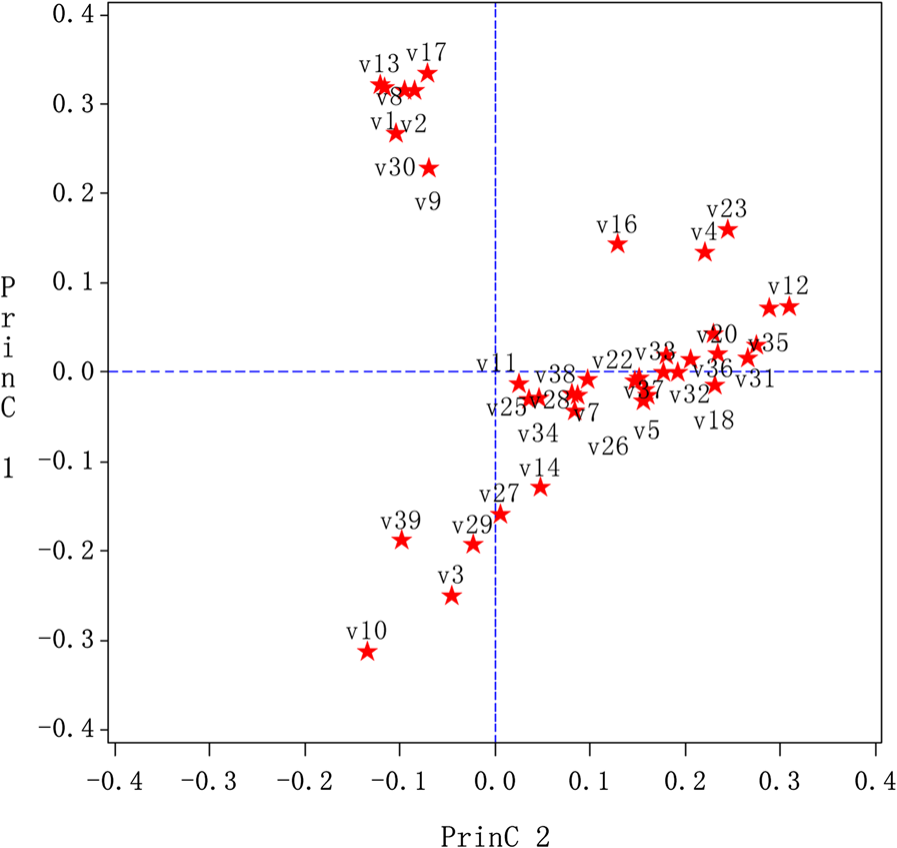
**Overall PCA loading plot of the GC-MS signals associated with the three species of Zhishi samples: 15 samples from *CJ*, 21 samples from *CJ *** **and 4 samples from *PT*.**

The corresponding PCA loading plot was utilized to identify the differential volatiles accountable for the separation among *CS*, *CJ* and *PT*. Although some information of low intensity might be lost due to Par scaling, the loading plot clearly facilitated the profiling of the volatiles in our experiments. The volatiles that accounted for maximum variance in the data set are given more weight or loading. As shown in Figure 3, the variation within PC2 in the loading plot was due to the marker volatiles that accounted for the differences between *PT* and the others as observed in the scores plot. Analogously, the distribution in PC1 of the loadings plot demonstrated the variation of *CS* and *CJ*, respectively. For example, sesquiterpenes, such as germacrene D (0.34 on PC2), caryophyllen (0.33 on PC2), β-myrcene (0.32 on PC2), β-elemene (0.31 on PC2) and α-farnesene (0.27 on PC2) were the most differentiating volatiles, which played an important role in differentiating *PT* from the *CS* and *CJ*. Meanwhile, in PC1, monoterpene compounds, such as p-mentha-1, 4(8)-diene (loading of 0.29), limonene (0.28), (+)-4-carene (0.28), β-pinene (0.28), γ-terpinene (0.28), α-thujene (0.27), p-cymen-2-ol (0.26) and α-pinene (0.26), appeared to be of some relevance to the observed variability between *CS* and *CJ*.

## Discussion

Literature reveals that limonene and γ-terpinene were the major components of the *Citrus* oils obtained by steam distillation and cold pressing [23]. The essential oils of four different species of Zhishi samples have been well studied and the major chemical groups are similar to the previous studies [21-23]. Limonene as a major constituent in several citrus oils (orange, lemon, mandarin, lime and grapefruit) is identified as the most predominant compound in the four species of Zhishi samples. The chemical compositions and their content of the essential oils are varied in different species. Especially, sesquiterpenes were present in the oils analyzed in the greatest amount in the *PT* species compared to the other three species. The contents of some monoterpene compounds such as p-mentha-1,4(8)-diene, limonene, (+)-4-carene, β-pinene, γ-terpinene, α-thujene, p-cymen-2-ol and α-pinene could be used as taxonomic markers between *CS* and *CJ*. The volatiles in *CA* could not be distinguished from the other three species. This may be because that *CA* samples distributed in a wide regions in China (Jiangxi, Hunan, Sichuan and Zhejiang). In the future, the active constituents contained in Zhishi samples from different regions need to be further investigated. Considering that all of the samples were collected in May or June under the same conditions impact of the growth stage factors on essential oil composition variability has been excluded.

### Experimental

#### Plant material

All of the 75 Zhishi samples were collected from the main Zhishi-producing provinces of China: Jiangxi, Sichuan, Zhejiang, Fujian and Guizhou in May or June during a three-year period. For each of the 75 Zhishi samples, the species, local name, collection location, year of collection and growing environment are listed in Table 2. The diameters of collected Zhishi samples were limited to 0.5 cm ~ 2.5 cm, which is consistent with the requirements of the Chinese Pharmacopoeia (Chinese Pharmacopoeia, 2010). Professors Ge Fei, Yan Zhuyun, Zhang Yungui, Xu Jianguo and Ke Fuzhi identified them as genuine samples of *C. aurantium* L. and its cultivars (*CA*), *C. sinensis* Osbeck and its cultivars (*CS*), *C. junos* Sieb. ex Tanaka (*CJ*) and Poncirus trifoliate Raf. (PT). The dried specimens (marked as *CA*-1 ~ *CA*-35, *CS*-1 ~ *CS*-21, *CJ*-1 ~ *CJ*-15 and *PT*-1 ~ *PT*-4) were deposited at the Institute of Basic Theory, China Academy of Chinese Medical Sciences, Beijing, P. R. China. These four *PT* samples were wild growing.

**Table 2:** The origins of the 75 Zhishi samples collected

**No.**	**Species**	**Local name**	**Location and collection time**	**Growing environment**
*Citrus aurantium* L.				
*CA*-1 ~ 3	*CA* cv *Xiucheng*	Xiucheng	Xingan, Jiangxi; 2011	Plain (N 27° E 115°; Alt.20 ~ 30 m)
*CA*-4 ~ 5	*CA* × *P. trifoliata*	Citrange	Yuanjiang, Hunan; 2010	Plain (N 28° E 112°; Alt.30 ~ 40 m)
*CA*-6 ~ 9	*CA*	Sour orange	Jiangjin, Sichuan; 2010-2012	Hillsides (N 29° E 106°; Alt.229 m)
*CA*-10	*CA* cv Daidai	Daidai	Jiangjin, Sichuan; 2012	Field margins (N 29° E 106°; Alt.200 ~ 230 m)
*CA*-11 ~ 19	*CA* cv *Xiucheng*	Xiucheng	Xingan, Jiangxi; 2012	Plain (N 27° E 115°; Alt.20 ~ 30 m)
*CA*-20 ~ 24	*CA* cv Xiucheng	Xiucheng	Zhangshu, Jiangxi; 2012	Plain (N 27° E 115°; Alt.20 ~ 30 m)
*CA*-25	*CA* cv Jizicheng	Jizicheng	Zhangshu, Jiangxi; 2012	Plain (N 27° E 115°; Alt.20 ~ 30 m)
*CA*-26	*CA* cv Daidai	Daidai	Huangyan, Zhejiang; 2012	Hillsides (N 28° E 121°; Alt.45 m)
*CA*-27	*CA* cv Morocco sour orange	Morocco sour orange	Huangyan, Zhejiang; 2012	Hillsides (N 28° E 121°; Alt.45 m)
*CA*-28 ~ 35	*CA* cv *Xiucheng*	Xiucheng	Xingan, Jiangxi; 2013	Plain (N 27° E 115°; Alt.20 ~ 30 m)
*Citurs junos* Sieb*.*ex Tanaka				
*CJ*-1**~**3	*CJ*	Xiangcheng	Xingan, Jiangxi; 2011	Hillsides (N 27° E 115°; Alt.50 ~ 60 m)
*CJ*-4	*CJ*	TuanyeXiangcheng	Jiangjin, Sichuan; 2012	Field margins (N 29° E 106°; Alt.200 ~ 230 m)
*CJ*-5 ~ 10	*CJ*	Xiangcheng	Xingan, Jiangxi; 2012	Hillsides (N 27° E 115°; Alt.50 ~ 60 m)
*CJ*-11 ~ 15	*CJ*	Xiangcheng	Xingan, Jiangxi; 2013	Hillsides (N 27° E 115°; Alt.50 ~ 60 m)
*Citrus sinensis* Osbeck				
*CS*-1	*CS* cv Jin Cheng	Jin Cheng	Qinglong, Guizhou; 2011	Hillsides (N 25° E 105°; Alt.1200 ~ 1300 m)
*CS*-2	*CS*	Navel Orange	Xingan, Jiangxi; 2011	Hillsides (N 27° E 115°; Alt.50 ~ 60 m)
*CS*-3	*CS* cv Jin Cheng	Jin Cheng	Qinglong, Guizhou; 2011	Hillsides (N 25° E 105°; Alt.1200 ~ 1300 m)
*CS*-4	*CS* cv Navel orange	Navel orange	Qinglong, Guizhou; 2011	Hillsides (N 25° E 105°; Alt.1200 ~ 1300 m)
*CS*-5	*CS* cv Blood orange	Blood orange	Qinglong, Guizhou; 2011	Hillsides (N 25° E 105°; Alt.1200 ~ 1300 m)
*CS*-6	*CS* cv Valencia	Valencia	Qinglong, Guizhou; 2011	Hillsides (N 25° E 105°; Alt.1200 ~ 1300 m)
*CS*-7	*CS* cv Blood orange	Blood orange	Jiangjin, Sichuan; 2012	Hillsides (N 29° E 106°; Alt.238 m)
*CS*-8	*CS* cv Valencia	Valencia	Jiangjin, Sichuan; 2012	Hillsides (N 29° E 106°; Alt.238 m)
*CS*-9	*CS* cv Navel orange	Navel orange	Jiangjin, Sichuan; 2012	Hillsides (N 29° E 106°; Alt.238 m)
*CS*-10	*CS* cv Peng an 100	Peng an 100	Jiangjin, Sichuan; 2012	Hillsides (N 29° E 106°; Alt.238 m)
*CS*-11 ~ 14	*CS* cv Liu Ben Cheng	Liu Ben Cheng	Huangyan, Zhejiang; 2012	Plain (N 28° E 121°; Alt.7 m)
*CS*-15	*CS* cv Hamlin	Hamlin	Huangyan, Zhejiang; 2012	Plain (N 28° E 121°; Alt.7 m)
*CS*-16	*CS* cv Jin Cheng	Jin Cheng	Huangyan, Zhejiang; 2012	Hillsides (N 28° E 121°; Alt.40 ~ 60 m)
*CS*-17	*CS* cv Navel orange	Navel orange	Huangyan, Zhejiang; 2012	Hillsides (N 28° E 121°; Alt.40 ~ 60 m)
*CS*-18 ~ 21	*CS* cv Delta Valencia	Delta Valencia	Huangyan, Zhejiang; 2012	Hillsides (N 28° E 121°; Alt.40 ~ 60 m)
*Poncirus trifoliate* Raf.				
*PT*-1	*PT*	Trifoliate orange	Jiangjin, Sichuan; 2012	Field margins (N 29° E 106°; Alt.200 ~ 230 m)
*PT*-2	*PT*	Trifoliate orange	Huangyan, Zhejiang; 2012	Hillsides (N 28° E 121°; Alt.45 m)
*PT*-3 ~ 4	*PT*	Trifoliate orange	Huangyan, Zhejiang; 2012	Hillsides (N 28° E 121°; Alt.45 m)

### Extraction of essential oils

Fresh, immature fruits of Zhishi were cut and dried in a manner similar to that described in the Pharmacopoeia of China [14]. The dried fruits were soaked in water for 2 hours and steam-distilled in a clevenger-type apparatus for 8 hours. This procedure was repeated 6 times. The oils were then dried over anhydrous sodium sulfate, diluted by acetone, and stored at 4–6°C before analysis.

### GC–MS analysis

GC–MS analysis was performed on GCMS-QP2010 Plus (Shimadzu, Kyoto) equipped with a capillary column (Rxi-5 ms, 30 m × 0.25 mm, 0.25 μm). Helium was used as the carrier gas at a flow rate of 1.0 mL/min. Oven temperature was varied from 50°C (1 min held) to 140°C(1 min held) at 5°C/min, and then from 140°C to 200°C (3 min held) at 10°C/min. The injector and interface temperatures were held at 250°C. Mass spectra in the electron impact mode (EI-MS) were generated at 70 eV. The ion source temperature was held at 250°C. The scanning time was 0.5 sec over a range of *m/z* 45–450. An oil sample of 1 μL was injected in the split mode injection (split ratio, 60:1).

### Identification and quantitative determination

The volatile components were tentatively identified based on linear retention index (RI) and by the comparison of mass spectra with MS data of reference compounds. The linear retention indices were determined for all constituents by using a homologous series of *n*-alkanes (C_8_–C_20_). The components were identified by comparison of their mass spectra with those of the NIST05 and NIST05S mass spectral library and further confirmed by comparison with the literature values [19,25]. The relative proportions of the essential oil constituents were expressed as percentages obtained by peak area normalization with all relative response factors being taken as one. The samples were run in triplicate.

### Statistical analysis

The GC-MS data of different species of Zhishi samples were analyzed to identify potential discriminant variables. Multivariate statistical analyses, including unsupervised PCA and supervised partial least squares-discriminant analysis (PLS-DA), were performed using the SAS 9.1.3 statistical package (order no. 195557). PCA was used to observe the natural interrelationship among the chemical components for each of the four *Citrus* species. Analysis of variance was employed to identify those volatiles that would be most differentiating among the four species. Furthermore, the corresponding loading plot was used to interpret the variations among the samples. The critical *p* value for all analyses in this study was set to 0.05.

## Conclusion

GC-MS coupled with multivariate statistical analysis of volatiles from different *Citrus* species, revealed a diverse volatile distribution among *CS*, *CJ* and *PT*. However, the volatiles contained in *CA* exhibited less specificity; therefore, they could not be separated from the other three species. The PCA scores plot of *CS*, *CJ* and *PT* appeared as widely distinct clustering. The chemical compounds accountable for the different volatile profiles of the three species are germacrene D, caryophyllen, β-myrcene, β-elemene, α-farnesene, p-mentha-1, 4(8)-diene, limonene, (+)-4-carene, β-pinene, γ-terpinene, α-thujene, p-cymen-2-ol and α-pinene, all of which are significantly up- or down- regulated in the different three species. In short, sesquiterpene compounds could serve as characteristic markers for *PT*. The content of some monoterpene compounds could be used as taxonomic markers between *CS* and *CJ*. This work demonstrates the variations of essential oils among different species of *Citrus* fruits and is of great significance in the pharmacological and clinical use of Zhishi.

## Abbreviations

GC-MS: Gas chromatography–mass spectrometry; CS: *Citrus sinensis*; CJ: *C. junos Sieb.* ex Tanaka; CA: *C. aurantium* L; PT: *Poncirus trifoliate* Raf; PCA: Principal component analysis; RI: Retention index; PLS-DA: Partial least squares-discriminant analysis; PC1: Principal component 1; PC2: Principal component 2; EI-MS: Electron impact mode.

## Competing interests

The authors declare that they have no competing interests.

## Authors’ contributions

AL provided the concept and designed the study. YL and ZL conducted the analyses and wrote the manuscript. CW, QZ, CL, ZS, ZN, SZ and XM participated in the research. All authors have read and approved the final manuscript.

## Supplementary Material

Additional file 1: Table S1 Identifications of Zhishi volatile in *Citrus sinensis* Osbeck and its cultivars. **Table S2.** Identifications of Zhishi volatile in *Citrus junos* Sieb. ex Tanaka. **Table S3.** Identifications of Zhishi volatile in *Poncirus aurantium* L. and its cultivars **Table S4.** Identifications of Zhishi volatile in *Poncirus trifoliate* Raf. **Table S5.** PCA loadings plot scores of all the GC-MS signals of the three species of Zhishi samples: 15 samples from *CJ*, 21 samples from *CJ* and 4 samples from *PT*. Click here for file
