# Supplementary material for: Study on essential oils from four species of Zhishi with gas chromatography–mass spectrometry
Source: Chem Cent J. 2014 Apr 3;8:22. doi: 10.1186/1752-153X-8-22 (PMC4234976; doi:10.1186/1752-153X-8-22)
Supplement: Additional file 1: Table S1 — Identifications of Zhishi volatile in Citrus sinensis Osbeck and its cultivars. Table S2. Identifications of Zhishi volatile in Citrus junos Sieb. ex Tanaka. Table S3. Identifications of Zhishi volatile in Poncirus aurantium L. and its cultivars Table S4. Identifications of Zhishi volatile in Poncirus trifoliate Raf. Table S5. PCA loadings plot scores of all the GC-MS signals of the three species of Zhishi samples: 15 samples from CJ, 21 samples from CJ and 4 samples from PT. [file 1752-153X-8-22-S1.doc]

**Supplementary Material**

**Study on essential oils from four species of *Citrus* fruits with gas chromatography-mass spectrometry**

Yuanyan Liu**^§^**^1^, Zhenli Liu**^§^**^2^, Chun Wang^2^, Qinglin Zha^3^, Cheng Lu^3^, Zhiqian Song^2^, Zhangchi Ning^1^, Siyu Zhao^1^, Xinmiao lu^1^, Aiping Lu3, 4[[1]](#footnote-2)*

**Affiliation**

^1^ School of Chinese Materia Medica, Beijing University of Chinese Medicine, Beijing Municipal Key Laboratory for Basic Research of Chinese Medicine, Beijing, 100102, China

^2^ Institution of Basic Theory, China Academy of Chinese Medical Sciences, Beijing, 100700, China

^3^ Institute of Basic Research in Clinical Medicine, China Academy of Chinese Medical Sciences, Beijing, 100700, China

^4^ School of Chinese Medicine, Hong Kong Baptist University, Hongkong, China

Table 1 Identifications of Zhishi volatile in *Citrus junos* Sieb. ex Tanaka

| Name | RI | CJ1 | CJ2 | CJ3 | CJ4 | CJ5 | CJ6 | CJ7 | CJ8 | CJ9 | CJ10 | CJ11 | CJ12 | CJ13 | CJ14 | CJ15 |
| --- | --- | --- | --- | --- | --- | --- | --- | --- | --- | --- | --- | --- | --- | --- | --- | --- |
| α-Thujene | 943 | 0.58 | 1.2 | 1.71 | 1.41 | 1.32 | 1.36 | 0.89 | 1.66 | 1.08 | 0.99 | 0.66 | 0.35 | 1.17 | 1.17 | 1.19 |
| α-Pinene | 948 | 1.41 | 2.67 | 3.97 | 3.39 | 3.02 | 3.13 | 2.27 | 3.76 | 2.72 | 2.46 | 1.48 | 0.88 | 2.6 | 2.64 | 2.74 |
| (+)-Sabinene | 979 | 0.55 | 0.29 | 0.31 | 1.26 | 0.43 | 0.47 | 2.21 | 0.54 | 0.36 | 0 | 0.36 | 0.15 | 0.41 | 0.34 | 0.39 |
| β-Pinene | 982 | 2.17 | 3.11 | 3.96 | 3.16 | 3.08 | 2.85 | 2.45 | 3.72 | 2.34 | 2.26 | 2.26 | 1.64 | 3.19 | 2.89 | 2.91 |
| β-Myrcene | 992 | 0.9 | 1.02 | 1.55 | 0.88 | 1.04 | 1.01 | 1.01 | 0.96 | 1.02 | 1.02 | 0.9 | 0.55 | 0.79 | 0.78 | 0.74 |
| n-Octanal | 1002 | 0 | 0 | 0 | 0.17 | 0.09 | 0.13 | 0.27 | 0.09 | 0.15 | 0.17 | 0.07 | 0 | 0 | 0.08 | 0.01 |
| α-Phellandrene | 1004 | 0.07 | 0.1 | 0.12 | 0.37 | 0.1 | 0.08 | 0.08 | 0.1 | 0.06 | 0.06 | 0.08 | 0.04 | 0.06 | 0.05 | 0.04 |
| (+)-4-Carene | 1015 | 0.88 | 1.1 | 1.23 | 0.8 | 0.94 | 0.82 | 0.69 | 1.03 | 0.54 | 0.62 | 1.06 | 0.84 | 1.06 | 0.83 | 0.75 |
| o-Cymene | 1022 | 4.96 | 2.73 | 2.78 | 1.87 | 1.08 | 0.48 | 0.45 | 0.93 | 1.06 | 0.62 | 0.55 | 0.38 | 0.85 | 1.52 | 2.34 |
| Limonene | 1026 | 35.53 | 37.62 | 52.11 | 42.25 | 48.18 | 52.3 | 57.43 | 42.96 | 60.6 | 60.34 | 38.46 | 39.14 | 37.23 | 46.43 | 46.51 |
| β-trans-Ocimene | 1033 | 0.57 | 0.36 | 0 | 0 | 0 | 0 | 0 | 0 | 0 | 0 | 0.03 | 0 | 0 | 0 | 0 |
| β-cis-Ocimene | 1042 | 0 | 0 | 0.36 | 0.89 | 0.28 | 0.51 | 0.66 | 0.34 | 0.52 | 0.54 | 0.75 | 0.26 | 0 | 0.31 | 0.25 |
| γ-Terpinen | 1052 | 35.04 | 39.59 | 25.6 | 32.95 | 33.92 | 31.2 | 21.71 | 35.66 | 25.5 | 26.09 | 41.88 | 46.74 | 44.73 | 36.05 | 35.74 |
| cis-β-Terpineol | 1060 | 0.09 | 0.05 | 0 | 0.13 | 0.03 | 0.06 | 0.09 | 0.07 | 0.01 | 0.05 | 0.06 | 0.04 | 0.01 | 0.02 | 0 |
| 1-Octanol | 1064 | 0 | 0 | 0 | 0 | 0 | 0 | 0 | 0 | 0 | 0 | 0 | 0 | 0 | 0 | 0 |
| α-Methyl-α-[4-methyl-3-pentenyl]oxiranemethanol | 1065 | 0.38 | 0.33 | 0.18 | 0 | 0.11 | 0.05 | 0.13 | 0.1 | 0.03 | 0.02 | 0.11 | 0 | 0.06 | 0.05 | 0.02 |
| p-Mentha-1,4 (8)-diene | 1081 | 2.06 | 2.11 | 2.24 | 1.26 | 1.48 | 1.23 | 0.97 | 1.55 | 0.95 | 1.1 | 2.33 | 2.15 | 2 | 1.52 | 1.46 |
| β-Linalool | 1089 | 1.63 | 2.49 | 0.73 | 7.95 | 3.32 | 2.91 | 7.36 | 4.83 | 1.98 | 2.03 | 4.46 | 4.3 | 2.89 | 3.31 | 2.8 |
| trans-1-methyl-4-(1-methylethyl)-2-Cyclohexen-1-ol | 1112 | 0 | 0.02 | 0 | 0 | 0 | 0 | 0 | 0 | 0 | 0 | 0 | 0 | 0 | 0 | 0 |
| cis-1-methyl-4-(1-methylethyl)-2  -Cyclohexen-1-ol | 1129 | 0 | 0 | 0 | 0 | 0 | 0 | 0 | 0 | 0 | 0 | 0 | 0 | 0 | 0 | 0 |
| β-Citronellal | 1141 | 0 | 0 | 0 | 0 | 0 | 0 | 0.03 | 0 | 0 | 0 | 0.05 | 0 | 0 | 0 | 0 |
| (-)-Terpinen-4-ol | 1164 | 1.02 | 0.95 | 0.67 | 0.33 | 0.37 | 0.37 | 0.39 | 0.47 | 0.3 | 0.31 | 0.53 | 0.68 | 0.35 | 0.27 | 0.21 |
| α-Terpieol | 1176 | 0.85 | 0.85 | 0.47 | 0.85 | 0.56 | 0.66 | 0.47 | 0.7 | 0.46 | 0.52 | 0.7 | 0.85 | 0.38 | 0.41 | 0.3 |
| n-Decanal | 1191 | 0 | 0 | 0.02 | 0 | 0 | 0 | 0.03 | 0 | 0 | 0 | 0.07 | 0 | 0 | 0.01 | 0 |
| cis-Carveol | 1202 | 0 | 0 | 0 | 0 | 0 | 0 | 0 | 0 | 0 | 0 | 0 | 0 | 0 | 0 | 0 |
| β-Citronellol | 1210 | 0 | 0 | 0.02 | 0 | 0 | 0 | 0 | 0 | 0 | 0 | 0.04 | 0 | 0 | 0 | 0 |
| β-Citral | 1224 | 0 | 0 | 0 | 0 | 0 | 0 | 0.04 | 0 | 0 | 0 | 0.02 | 0 | 0 | 0 | 0 |
| trans-Geraniol | 1236 | 0 | 0 | 0 | 0 | 0 | 0 | 0 | 0 | 0 | 0 | 0 | 0 | 0 | 0 | 0 |
| α-Citral | 1251 | 0 | 0 | 0 | 0 | 0 | 0 | 0.03 | 0 | 0 | 0 | 0.03 | 0 | 0 | 0.01 | 0 |
| p-Cymen-2-ol | 1269 | 1 | 0.86 | 1.23 | 0 | 0.54 | 0.28 | 0.26 | 0.53 | 0.26 | 0.33 | 0.87 | 0.21 | 0.73 | 0.47 | 0.63 |
| Nerol acetate | 1334 | 0 | 0 | 0 | 0 | 0 | 0 | 0 | 0 | 0 | 0 | 0 | 0 | 0 | 0 | 0 |
| β-Elemene | 1362 | 0 | 0 | 0 | 0 | 0 | 0 | 0 | 0 | 0 | 0 | 0 | 0 | 0 | 0 | 0 |
| Caryophyllene | 1387 | 0 | 0 | 0 | 0 | 0 | 0 | 0 | 0 | 0 | 0 | 0 | 0 | 0 | 0 | 0 |
| β-Farnesene | 1418 | 0.15 | 0.08 | 0.09 | 0 | 0 | 0 | 0 | 0 | 0 | 0 | 0.07 | 0 | 0 | 0 | 0 |
| Germacrene D | 1442 | 0.55 | 0.34 | 0.26 | 0 | 0.11 | 0.1 | 0.07 | 0 | 0.06 | 0.08 | 0.35 | 0.09 | 0.06 | 0.09 | 0.02 |
| 4-isopropylidene-1-vinyl-o-Menth-8-ene | 1452 | 0.05 | 0.03 | 0 | 0 | 0 | 0 | 0 | 0 | 0 | 0 | 0.02 | 0 | 0 | 0 | 0 |
| (Z,E)-α-Farnesene | 1462 | 0 | 0 | 0 | 0.08 | 0 | 0 | 0 | 0 | 0 | 0 | 0 | 0 | 0 | 0 | 0 |
| γ-Elemene | 1493 | 0 | 0 | 0 | 0 | 0 | 0 | 0 | 0 | 0 | 0 | 0 | 0 | 0 | 0 | 0 |
| α-Sinensal | 1567 | 0 | 0 | 0 | 0 | 0 | 0 | 0 | 0 | 0 | 0 | 0 | 0 | 0 | 0 | 0 |

Table 2 Identifications of Zhishi volatile in *Citrus aurantium* L*.* and its cultivars.

| Name | RI | CA1 | CA2 | CA3 | CA4 | CA5 | CA6 | CA7 | CA8 | CA9 | CA10 | CA11 | CA12 |
| --- | --- | --- | --- | --- | --- | --- | --- | --- | --- | --- | --- | --- | --- |
| α-Thujene | 943 | 0.21 | 0.99 | 0.76 | 1.11 | 0.47 | 0.04 | 0.05 | 0.04 | 0 | 0 | 0.54 | 0.57 |
| α-Pinene | 948 | 0.51 | 2.45 | 2.71 | 2.69 | 1.28 | 0.68 | 1.44 | 1.11 | 0.8 | 0.52 | 1.44 | 1.57 |
| (+)-Sabinene | 979 | 6.68 | 1.82 | 2.11 | 0.16 | 0.19 | 0.52 | 2.8 | 2.08 | 2.13 | 0.91 | 0.28 | 0.62 |
| β-Pinene | 982 | 0.68 | 2.35 | 0.54 | 2.75 | 1.32 | 6.37 | 17.01 | 12.99 | 10.17 | 3 | 1.16 | 1.23 |
| β-Myrcene | 992 | 1.2 | 1.97 | 0.56 | 0.87 | 0.87 | 0.64 | 2.72 | 1.77 | 0.97 | 1.22 | 1.07 | 1.08 |
| n-Octanal | 1002 | 0.16 | 0.23 | 0 | 0 | 0 | 0 | 0.87 | 0.63 | 0.43 | 0.09 | 0.73 | 0.41 |
| α-Phellandrene | 1004 | 0.05 | 0.1 | 0 | 0.07 | 0.03 | 0 | 0.07 | 0.04 | 0 | 0.03 | 0.09 | 0.07 |
| (+)-4-Carene | 1015 | 0.45 | 0.84 | 0.16 | 0.75 | 0.26 | 0.12 | 0.26 | 0.15 | 0.05 | 0.04 | 0.37 | 0.38 |
| o-Cymene | 1022 | 0.17 | 1.04 | 5.86 | 1.84 | 5.01 | 0.12 | 0 | 0 | 0.07 | 0.03 | 0.34 | 0.14 |
| Limonene | 1026 | 44.76 | 39.28 | 40.53 | 27 | 57.32 | 72.4 | 28.89 | 47.18 | 70.79 | 68.44 | 72.94 | 71.64 |
| β-trans-Ocimene | 1033 | 0.1 | 0.1 | 0 | 0.29 | 0.04 | 0.31 | 0.23 | 0.13 | 0.04 | 0.18 | 0 | 0 |
| β-cis-Ocimene | 1042 | 3.63 | 4.52 | 0.14 | 9.88 | 1.27 | 0 | 9.99 | 6.14 | 4.79 | 3.35 | 0.59 | 1.37 |
| γ-Terpinen | 1052 | 3.14 | 25.46 | 15.99 | 28.65 | 10.07 | 0.25 | 0.41 | 0.26 | 0.85 | 0.45 | 14.7 | 14.75 |
| cis-β-Terpineol | 1060 | 0.52 | 0.05 | 0 | 0 | 0 | 0 | 0.09 | 0.07 | 0.06 | 0 | 0.05 | 0.03 |
| 1-Octanol | 1064 | 0.03 | 0.02 | 0 | 0 | 0 | 0 | 0.08 | 0.07 | 0.1 | 0.02 | 0.02 | 0.02 |
| α-Methyl-α-[4-methyl-3-pentenyl]oxiranemethanol | 1065 | 0.18 | 1.18 | 0.15 | 1.03 | 1.26 | 0.33 | 0.64 | 0.27 | 0.13 | 0.4 | 0.33 | 0.25 |
| p-Mentha-1,4(8)-diene | 1081 | 0.47 | 1.79 | 0.28 | 1.77 | 1.21 | 0.26 | 0.46 | 0.2 | 0.12 | 0.31 | 0.74 | 0.67 |
| β-Linalool | 1089 | 26.15 | 10.83 | 10.81 | 9.36 | 14 | 4.98 | 25.25 | 21.23 | 7.24 | 18.29 | 2.93 | 3.74 |
| trans-1-methyl-4-(1-methylethyl)-2-Cyclohexen-1-ol | 1112 | 1.1 | 0.05 | 0 | 0 | 0 | 0.3 | 0.08 | 0.04 | 0 | 0 | 0 | 0 |
| cis-1-methyl-4-(1-methylethyl)-2-Cyclohexen-1-ol | 1129 | 0.07 | 0.02 | 0 | 0 | 0 | 0 | 0.04 | 0 | 0 | 0 | 0 | 0 |
| β-Citronellal | 1141 | 0.2 | 0.12 | 0 | 0 | 0 | 0 | 0.07 | 0.04 | 0 | 0 | 0.04 | 0.11 |
| (-)-Terpinen-4-ol | 1164 | 3.23 | 1.57 | 2.58 | 0.41 | 0.51 | 1.2 | 1.9 | 1.15 | 0.08 | 0.06 | 0.24 | 0.19 |
| α-Terpieol | 1176 | 1.08 | 1.2 | 0.35 | 0.53 | 0.81 | 1.15 | 1.93 | 1.19 | 0.24 | 0.94 | 0.58 | 0.34 |
| n-Decanal | 1191 | 0.07 | 0.14 | 0 | 0 | 0 | 0 | 0.52 | 0.29 | 0.11 | 0 | 0.07 | 0.06 |
| cis-Carveol | 1202 | 0 | 0 | 0 | 0 | 0.07 | 0.95 | 0.03 | 0 | 0 | 0 | 0 | 0 |
| β-Citronellol | 1210 | 0.06 | 0 | 0 | 0 | 0 | 0.44 | 0 | 0 | 0.08 | 0.08 | 0 | 0.12 |
| β-Citral | 1224 | 0 | 0.03 | 0 | 0 | 0.05 | 0.72 | 0.05 | 0.02 | 0 | 0.03 | 0.09 | 0.09 |
| trans-Geraniol | 1236 | 0 | 0 | 0 | 0.04 | 0 | 0 | 0.11 | 0.04 | 0 | 1.16 | 0 | 0 |
| α-Citral | 1251 | 0 | 0 | 0 | 0 | 0 | 0 | 0.07 | 0 | 0 | 0.04 | 0.09 | 0.08 |
| p-Cymen-2-ol | 1269 | 0 | 0.04 | 1.29 | 0.78 | 0.89 | 0 | 0 | 0 | 0 | 0 | 0 | 0 |
| Nerol acetate | 1334 | 0.1 | 0.06 | 0.04 | 0 | 0 | 0 | 0.17 | 0.1 | 0 | 0.09 | 0 | 0.03 |
| β-Elemene | 1362 | 0 | 0 | 0 | 0.1 | 0 | 0 | 0.03 | 0 | 0 | 0.21 | 0 | 0 |
| Caryophyllene | 1387 | 0 | 0 | 0 | 0 | 0 | 0 | 0 | 0 | 0 | 0 | 0 | 0 |
| β-Farnesene | 1418 | 0 | 0 | 0 | 0 | 0 | 0 | 0 | 0 | 0 | 0 | 0 | 0 |
| Germacrene D | 1442 | 0.21 | 0.42 | 0.28 | 0.44 | 0.13 | 0.48 | 0.99 | 0.87 | 0.48 | 0.04 | 0.37 | 0.2 |
| 4-isopropylidene-1-vinyl-o-Menth-8-ene | 1452 | 0 | 0.03 | 0 | 0.05 | 0 | 0 | 0.08 | 0.07 | 0 | 0 | 0 | 0 |
| (Z,E)-α-Farnesene | 1462 | 0 | 0.02 | 0 | 0 | 0.05 | 0 | 0.02 | 0 | 0 | 0 | 0 | 0 |
| γ-Elemene | 1493 | 0 | 0 | 0 | 0 | 0 | 0 | 0.19 | 0 | 0 | 0 | 0 | 0 |
| α-Sinensal | 1567 | 0 | 0 | 0 | 0 | 0 | 0 | 0 | 0 | 0 | 0 | 0 | 0 |

Continues:

| Name | RI | CA13 | CA14 | CA15 | CA16 | CA17 | CA18 | CA19 | CA20 | CA21 | CA22 | CA23 | CA24 |
| --- | --- | --- | --- | --- | --- | --- | --- | --- | --- | --- | --- | --- | --- |
| α-Thujene | 943 | 0.08 | 0.5 | 1.15 | 0.1 | 0.44 | 0.61 | 0.34 | 0.49 | 0.06 | 0.05 | 0.04 | 0.71 |
| α-Pinene | 948 | 0.58 | 1.35 | 2.97 | 0.67 | 1.12 | 1.62 | 1 | 1.27 | 0.65 | 0.56 | 0.57 | 1.81 |
| (+)-Sabinene | 979 | 3.28 | 0.29 | 1.09 | 5.74 | 0.23 | 0.27 | 0.21 | 0.38 | 3.6 | 3.54 | 4.16 | 0.29 |
| β-Pinene | 982 | 0.46 | 1.2 | 6.64 | 0.77 | 1.18 | 1.39 | 0.88 | 1.05 | 0.43 | 0.4 | 0.4 | 1.36 |
| β-Myrcene | 992 | 1.08 | 0.95 | 0.93 | 1.08 | 0.95 | 1.01 | 1.14 | 1.3 | 1.19 | 1.14 | 1.17 | 1.13 |
| n-Octanal | 1002 | 0.58 | 0.37 | 0 | 0.54 | 0.54 | 0.54 | 0.18 | 0.53 | 0.55 | 0.56 | 0.48 | 0.68 |
| α-Phellandrene | 1004 | 0.06 | 0.08 | 0.42 | 0.07 | 0.08 | 0.08 | 0.05 | 0.06 | 0.06 | 0 | 0 | 0.07 |
| (+)-4-Carene | 1015 | 0.12 | 0.37 | 0.74 | 0.19 | 0.44 | 0.46 | 0.26 | 0.38 | 0.12 | 0.14 | 0.11 | 0.46 |
| o-Cymene | 1022 | 0.05 | 0.31 | 0.62 | 0.07 | 0.2 | 0.32 | 0.19 | 0.79 | 0 | 0 | 0 | 0.53 |
| Limonene | 1026 | 76.75 | 70.58 | 50.46 | 72.93 | 67.15 | 65.92 | 82.84 | 67.95 | 79.03 | 77.76 | 78.2 | 68.78 |
| β-trans-Ocimene | 1033 | 0 | 0 | 0 | 0 | 0 | 0 | 0 | 0.04 | 0 | 0 | 0 | 0 |
| β-cis-Ocimene | 1042 | 1.12 | 1.59 | 1.01 | 1.24 | 1.62 | 2.5 | 0.23 | 1.15 | 0.92 | 0.85 | 0.94 | 1 |
| γ-Terpinen | 1052 | 2 | 16.09 | 28.36 | 2.17 | 18.36 | 17.94 | 10.2 | 12.71 | 0.67 | 0.64 | 0.41 | 14.42 |
| cis-β-Terpineol | 1060 | 0.09 | 0.02 | 0.02 | 0.18 | 0.03 | 0.03 | 0.02 | 0 | 0.08 | 0 | 0.1 | 0 |
| 1-Octanol | 1064 | 0 | 0 | 0 | 0.03 | 0.01 | 0.03 | 0 | 0.07 | 0 | 0 | 0 | 0.21 |
| α-Methyl-α-[4-methyl-3-pentenyl]oxiranemethanol | 1065 | 0.14 | 0.22 | 0.04 | 0.07 | 0.33 | 0.31 | 0.6 | 0.5 | 0.2 | 0.17 | 0.15 | 0.45 |
| p-Mentha-1,4(8)-diene | 1081 | 0.17 | 0.74 | 1.14 | 0.16 | 0.88 | 0.88 | 0.65 | 0.85 | 0.16 | 0.13 | 0.1 | 0.79 |
| β-Linalool | 1089 | 12.62 | 3.75 | 2.64 | 13.06 | 4.61 | 4.53 | 0.36 | 6.75 | 11.46 | 13.11 | 12.19 | 5.24 |
| trans-1-methyl-4-(1-methylethyl)-2-Cyclohexen-1-ol | 1112 | 0 | 0 | 0 | 0 | 0 | 0 | 0 | 0 | 0 | 0 | 0 | 0 |
| cis-1-methyl-4-(1-methylethyl)-2-Cyclohexen-1-ol | 1129 | 0 | 0 | 0 | 0 | 0 | 0 | 0 | 0 | 0 | 0 | 0 | 0 |
| β-Citronellal | 1141 | 0 | 0.13 | 0 | 0 | 0.13 | 0.13 | 0.05 | 0.19 | 0 | 0 | 0 | 0.06 |
| (-)-Terpinen-4-ol | 1164 | 0.25 | 0.24 | 0.52 | 0.31 | 0.28 | 0.28 | 0.1 | 0.6 | 0.25 | 0.4 | 0.33 | 0.4 |
| α-Terpieol | 1176 | 0.29 | 0.44 | 0.62 | 0.29 | 0.5 | 0.49 | 0.34 | 0.98 | 0.26 | 0.33 | 0.28 | 0.65 |
| n-Decanal | 1191 | 0 | 0 | 0 | 0.05 | 0.07 | 0.09 | 0 | 0.14 | 0.05 | 0 | 0.07 | 0.08 |
| cis-Carveol | 1202 | 0 | 0 | 0 | 0 | 0 | 0 | 0 | 0 | 0 | 0 | 0 | 0 |
| β-Citronellol | 1210 | 0.08 | 0.12 | 0 | 0.09 | 0.1 | 0.14 | 0 | 0.48 | 0.09 | 0 | 0.08 | 0.15 |
| β-Citral | 1224 | 0.07 | 0 | 0 | 0.05 | 0.07 | 0.06 | 0.03 | 0.3 | 0.08 | 0 | 0.05 | 0.12 |
| trans-Geraniol | 1236 | 0 | 0 | 0 | 0 | 0 | 0 | 0 | 0 | 0 | 0 | 0 | 0 |
| α-Citral | 1251 | 0.06 | 0 | 0 | 0.05 | 0.06 | 0.06 | 0.03 | 0.35 | 0.09 | 0.06 | 0 | 0.13 |
| p-Cymen-2-ol | 1269 | 0 | 0 | 0 | 0 | 0 | 0 | 0 | 0 | 0 | 0 | 0 | 0 |
| Nerol acetate | 1334 | 0 | 0 | 0.07 | 0 | 0 | 0 | 0 | 0.13 | 0 | 0 | 0 | 0 |
| β-Elemene | 1362 | 0 | 0 | 0 | 0 | 0 | 0 | 0.03 | 0 | 0 | 0 | 0 | 0 |
| Caryophyllene | 1387 | 0 | 0 | 0 | 0 | 0 | 0 | 0 | 0 | 0 | 0 | 0 | 0 |
| β-Farnesene | 1418 | 0 | 0 | 0 | 0 | 0 | 0 | 0 | 0 | 0 | 0 | 0 | 0 |
| Germacrene D | 1442 | 0.07 | 0.35 | 0.45 | 0.09 | 0.27 | 0.22 | 0.06 | 0.29 | 0 | 0.16 | 0.07 | 0.08 |
| 4-isopropylidene-1-vinyl-o-Menth-8-ene | 1452 | 0 | 0 | 0.05 | 0 | 0 | 0 | 0 | 0 | 0 | 0 | 0 | 0 |
| (Z,E)-α-Farnesene | 1462 | 0 | 0 | 0 | 0 | 0 | 0 | 0 | 0.02 | 0 | 0 | 0 | 0 |
| γ-Elemene | 1493 | 0 | 0 | 0 | 0 | 0 | 0 | 0 | 0 | 0 | 0 | 0 | 0 |
| α-Sinensal | 1567 | 0 | 0 | 0 | 0 | 0 | 0 | 0 | 0 | 0 | 0 | 0 | 0 |

Continues:

| Name | RI | CA25 | CA26 | CA27 | CA28 | CA29 | CA30 | CA31 | CA32 | CA33 | CA34 | CA35 |
| --- | --- | --- | --- | --- | --- | --- | --- | --- | --- | --- | --- | --- |
| α-Thujene | 943 | 0.51 | 0 | 1.12 | 0.07 | 0.32 | 0.57 | 0.22 | 0.02 | 0.5 | 0.86 | 0.43 |
| α-Pinene | 948 | 1.53 | 0.35 | 2.45 | 0.58 | 0.82 | 1.43 | 0.6 | 0.28 | 1.2 | 1.96 | 1.2 |
| (+)-Sabinene | 979 | 0.26 | 0.58 | 0.74 | 8.26 | 0.18 | 0.22 | 0.1 | 7.64 | 0.19 | 0.21 | 0.12 |
| β-Pinene | 982 | 0.99 | 1.31 | 3.8 | 0.53 | 0.95 | 1.38 | 0.82 | 0.4 | 1.52 | 2.06 | 1.04 |
| β-Myrcene | 992 | 1.26 | 0.98 | 0.91 | 1.12 | 0.73 | 0.76 | 0.52 | 0.87 | 0 | 0.71 | 0.84 |
| n-Octanal | 1002 | 0.29 | 0.07 | 0.05 | 0.24 | 0.7 | 0.1 | 0.14 | 0.26 | 0.33 | 0.24 | 0 |
| α-Phellandrene | 1004 | 0.06 | 0 | 0.08 | 0.02 | 0.02 | 0.02 | 0 | 0 | 0.03 | 0.04 | 0.02 |
| (+)-4-Carene | 1015 | 0.27 | 0 | 0.88 | 0.22 | 0.33 | 0.35 | 0.29 | 0.12 | 0.52 | 0.64 | 0.25 |
| o-Cymene | 1022 | 0.13 | 0 | 0.8 | 0.05 | 0.29 | 1.48 | 0.28 | 0.01 | 0.19 | 0.51 | 0.46 |
| Limonene | 1026 | 82.83 | 75.13 | 44.69 | 69.28 | 68.6 | 66.1 | 64.14 | 65.25 | 54.55 | 51.99 | 79.45 |
| β-trans-Ocimene | 1033 | 0 | 0.09 | 0 | 0 | 0.66 | 0 | 0 | 0 | 0.02 | 0.02 | 0 |
| β-cis-Ocimene | 1042 | 0.15 | 3.07 | 1.02 | 2.17 | 0 | 1.57 | 2.28 | 1.93 | 2.85 | 2.43 | 0.17 |
| γ-Terpinen | 1052 | 9.21 | 0.47 | 32.31 | 1.78 | 18.48 | 20.72 | 23.11 | 1.1 | 28.16 | 31.16 | 14.79 |
| cis-β-Terpineol | 1060 | 0.01 | 0 | 0.07 | 0.23 | 0.02 | 0 | 0 | 0.17 | 0.02 | 0 | 0 |
| 1-Octanol | 1064 | 0.01 | 0.05 | 0 | 0 | 0 | 0 | 0 | 0 | 0 | 0 | 0 |
| α-Methyl-α-[4-methyl-3-pentenyl]oxiranemethanol | 1065 | 0.79 | 0.62 | 0.25 | 0.06 | 0.15 | 0.22 | 0.1 | 0.05 | 0.26 | 0.27 | 0.09 |
| p-Mentha-1,4(8)-diene | 1081 | 0.69 | 0.34 | 1.45 | 0.13 | 0.79 | 0.85 | 0.86 | 0.07 | 1.18 | 1.29 | 0.55 |
| β-Linalool | 1089 | 0.32 | 15.41 | 7.52 | 12.32 | 3.66 | 2.5 | 3.95 | 18.82 | 5.17 | 3.72 | 0 |
| trans-1-methyl-4-(1-methylethyl)-2-Cyclohexen-1-ol | 1112 | 0 | 0 | 0 | 0 | 0 | 0 | 0 | 0 | 0 | 0 | 0 |
| cis-1-methyl-4-(1-methylethyl)-2-Cyclohexen-1-ol | 1129 | 0 | 0 | 0 | 0 | 0 | 0 | 0 | 0 | 0 | 0 | 0 |
| β-Citronellal | 1141 | 0.03 | 0 | 0 | 0 | 0 | 0.02 | 0.06 | 0.03 | 0 | 0 | 0 |
| (-)-Terpinen-4-ol | 1164 | 0.14 | 0.05 | 0.44 | 1.41 | 0.32 | 0.2 | 0.24 | 1.02 | 0.28 | 0.29 | 0 |
| α-Terpieol | 1176 | 0.34 | 0.73 | 0.6 | 0.36 | 0.85 | 0.33 | 0.43 | 0.47 | 0.67 | 0.5 | 0 |
| n-Decanal | 1191 | 0.03 | 0 | 0 | 0 | 0.13 | 0 | 0 | 0 | 0.05 | 0 | 0 |
| cis-Carveol | 1202 | 0 | 0 | 0 | 0 | 0 | 0 | 0 | 0 | 0 | 0 | 0 |
| β-Citronellol | 1210 | 0 | 0 | 0 | 0.03 | 0 | 0.02 | 0 | 0.08 | 0.03 | 0 | 0 |
| β-Citral | 1224 | 0.03 | 0 | 0 | 0 | 0.04 | 0.03 | 0 | 0 | 0.01 | 0 | 0 |
| trans-Geraniol | 1236 | 0 | 0.54 | 0 | 0 | 0 | 0 | 0 | 0 | 0 | 0 | 0 |
| α-Citral | 1251 | 0 | 0 | 0 | 0.02 | 0.08 | 0.01 | 0.03 | 0.03 | 0 | 0 | 0 |
| p-Cymen-2-ol | 1269 | 0 | 0 | 0.73 | 0 | 0 | 0 | 0 | 0 | 0 | 0 | 0 |
| Nerol acetate | 1334 | 0.03 | 0.11 | 0 | 0.05 | 0.04 | 0 | 0 | 0 | 0.02 | 0 | 0 |
| β-Elemene | 1362 | 0 | 0 | 0 | 0 | 0 | 0 | 0 | 0 | 0 | 0 | 0 |
| Caryophyllene | 1387 | 0 | 0 | 0 | 0 | 0 | 0 | 0 | 0 | 0 | 0 | 0 |
| β-Farnesene | 1418 | 0 | 0 | 0 | 0 | 0 | 0 | 0 | 0 | 0 | 0 | 0 |
| Germacrene D | 1442 | 0.06 | 0 | 0.09 | 0.12 | 0.77 | 0.19 | 0.14 | 0.17 | 0.51 | 0.09 | 0 |
| 4-isopropylidene-1-vinyl-o-Menth-8-ene | 1452 | 0 | 0 | 0 | 0 | 0.02 | 0 | 0 | 0 | 0 | 0 | 0 |
| (Z,E)-α-Farnesene | 1462 | 0 | 0 | 0 | 0 | 0 | 0 | 0 | 0 | 0.02 | 0 | 0 |
| γ-Elemene | 1493 | 0 | 0 | 0 | 0 | 0 | 0 | 0 | 0 | 0 | 0 | 0 |
| α-Sinensal | 1567 | 0 | 0 | 0 | 0 | 0 | 0 | 0 | 0 | 0 | 0 | 0 |

Table 3 Identifications of Zhishi volatile in *Poncirus trifoliate* Raf..

| Name | RI | PT1 | PT2 | PT3 | PT4 |
| --- | --- | --- | --- | --- | --- |
| α-Thujene | 943 | 0 | 0 | 0 | 0 |
| α-Pinene | 948 | 0 | 1.52 | 0.49 | 0.29 |
| (+)-Sabinene | 979 | 0.55 | 1.69 | 0.62 | 2.54 |
| β-Pinene | 982 | 0 | 1.64 | 3.46 | 2.2 |
| β-Myrcene | 992 | 21.85 | 8.6 | 28.91 | 13.8 |
| n-Octanal | 1002 | 0 | 0 | 0 | 0 |
| α-Phellandrene | 1004 | 0.84 | 9 | 6.36 | 2.75 |
| (+)-4-Carene | 1015 | 0 | 0.09 | 0.18 | 0.1 |
| o-Cymene | 1022 | 0 | 0.23 | 0.22 | 0 |
| Limonene | 1026 | 59.96 | 69.19 | 42.22 | 21.19 |
| β-trans-Ocimene | 1033 | 0 | 0 | 0.04 | 0.57 |
| β-cis-Ocimene | 1042 | 1.46 | 1.96 | 4.96 | 2 |
| γ-Terpinen | 1052 | 0 | 0.46 | 0.73 | 0.4 |
| cis-β-Terpineol | 1060 | 0 | 0 | 0 | 0 |
| 1-Octanol | 1064 | 0 | 0.03 | 0 | 0 |
| α-Methyl-α-[4-methyl-3-pentenyl]oxiranemethanol | 1065 | 0 | 0 | 0 | 0 |
| p-Mentha-1,4(8)-diene | 1081 | 0 | 0.07 | 0.12 | 0 |
| β-Linalool | 1089 | 0.37 | 0.7 | 0.37 | 1.18 |
| trans-1-methyl-4-(1-methylethyl)-2-Cyclohexen-1-ol | 1112 | 0 | 0.05 | 0 | 0.21 |
| cis-1-methyl-4-(1-methylethyl)-2-Cyclohexen-1-ol | 1129 | 0 | 0 | 0 | 0.12 |
| β-Citronellal | 1141 | 0 | 0 | 0 | 0 |
| (-)-Terpinen-4-ol | 1164 | 0 | 0.28 | 0.81 | 2.42 |
| α-Terpieol | 1176 | 0 | 0.58 | 0.31 | 2.1 |
| n-Decanal | 1191 | 0 | 0 | 0 | 0 |
| cis-Carveol | 1202 | 0 | 0 | 0 | 0 |
| β-Citronellol | 1210 | 0 | 0 | 0 | 0.3 |
| β-Citral | 1224 | 0 | 0 | 0 | 0 |
| trans-Geraniol | 1236 | 0 | 0 | 0 | 0 |
| α-Citral | 1251 | 0 | 0 | 0 | 0 |
| p-Cymen-2-ol | 1269 | 0 | 0 | 0 | 0 |
| Nerol acetate | 1334 | 0 | 0 | 0 | 0.55 |
| β-Elemene | 1362 | 0 | 0 | 0.13 | 0.96 |
| Caryophyllene | 1387 | 0 | 2.62 | 3.96 | 14.7 |
| β-Farnesene | 1418 | 8.94 | 0.32 | 0.22 | 1.71 |
| Germacrene D | 1442 | 0.64 | 0.49 | 1.44 | 4.75 |
| 4-isopropylidene-1-vinyl-o-Menth-8-ene | 1452 | 0 | 0 | 0.05 | 0.25 |
| (Z,E)-α-Farnesene | 1462 | 0 | 0 | 0 | 1.31 |
| γ-Elemene | 1493 | 1.9 | 0.37 | 2.35 | 10.6 |
| α-Sinensal | 1567 | 0 | 0 | 0 | 0 |

Table 4 Identifications of Zhishi volatile in *Citrus sinensis* Osbeck and its cultivars.

| Name | RI | CS1 | CS2 | CS3 | CS4 | CS5 | CS6 | CS7 | CS8 | CS9 | CS10 | CS11 |
| --- | --- | --- | --- | --- | --- | --- | --- | --- | --- | --- | --- | --- |
| α-Thujene | 943 | 0.21 | 0.05 | 0 | 0 | 0.06 | 0 | 0 | 0 | 0.06 | 0.03 | 0.04 |
| α-Pinene | 948 | 1 | 0.47 | 0.35 | 0.44 | 0.71 | 0.47 | 0.36 | 0.28 | 0.71 | 0.49 | 0.46 |
| (+)-Sabinene | 979 | 0.19 | 5.1 | 5 | 6.67 | 5.44 | 6.93 | 5.15 | 3.61 | 8.64 | 6.26 | 9.02 |
| β-Pinene | 982 | 0.86 | 0.42 | 0.35 | 0.49 | 0.54 | 0.55 | 0.53 | 0.42 | 0.79 | 0.78 | 0.72 |
| β-Myrcene | 992 | 0.85 | 1.14 | 1.09 | 1.11 | 1.2 | 1.12 | 1.03 | 0.96 | 1.34 | 1.15 | 1.29 |
| n-Octanal | 1002 | 0 | 0.14 | 0 | 0.17 | 0 | 0.15 | 0.19 | 0.22 | 0.05 | 0.1 | 0.07 |
| α-Phellandrene | 1004 | 0.08 | 0 | 0 | 0 | 0 | 0 | 0 | 0 | 0.04 | 0.03 | 0.04 |
| (+)-4-Carene | 1015 | 0.3 | 0.09 | 0.09 | 0.1 | 0.11 | 0.13 | 0.11 | 0.15 | 0.22 | 0.12 | 0.16 |
| o-Cymene | 1022 | 5.97 | 0 | 0 | 0 | 0.04 | 0 | 0 | 0.22 | 0 | 0.03 | 0 |
| Limonene | 1026 | 66.59 | 86.01 | 85.04 | 82.68 | 86.65 | 84.44 | 87.41 | 84.31 | 82 | 83.68 | 82.38 |
| β-trans-Ocimene | 1033 | 0.09 | 0 | 0 | 0 | 0 | 0.81 | 0 | 0 | 0 | 0.25 | 0 |
| β-cis-Ocimene | 1042 | 0 | 0.16 | 0.29 | 0.25 | 0.24 | 0 | 0.29 | 0.84 | 0.34 | 0 | 0.23 |
| γ-Terpinen | 1052 | 4.56 | 0.64 | 0.76 | 0.46 | 0.4 | 0.59 | 0.81 | 2.63 | 0.46 | 0.52 | 0.49 |
| cis-β-Terpineol | 1060 | 0 | 0.11 | 0 | 0.15 | 0 | 0 | 0.15 | 0.15 | 0.1 | 0.11 | 0.17 |
| 1-Octanol | 1064 | 0 | 0 | 0 | 0 | 0 | 0 | 0 | 0 | 0 | 0 | 0 |
| α-Methyl-α-[4-methyl-3-pentenyl]oxiranemethanol | 1065 | 0 | 0 | 0 | 0 | 0 | 0 | 0 | 0 | 0 | 0 | 0 |
| p-Mentha-1,4(8)-diene | 1081 | 0.41 | 0 | 0.05 | 0.05 | 0.04 | 0 | 0.07 | 0.15 | 0.08 | 0.07 | 0.07 |
| β-Linalool | 1089 | 1.3 | 4.92 | 6.02 | 6.05 | 3.86 | 3.75 | 2.53 | 4.22 | 3.95 | 5.3 | 3.57 |
| trans-1-methyl-4-(1-methylethyl)-2-Cyclohexen-1-ol | 1112 | 0.16 | 0 | 0 | 0 | 0 | 0 | 0 | 0 | 0 | 0 | 0 |
| cis-1-methyl-4-(1-methylethyl)-2-Cyclohexen-1-ol | 1129 | 0 | 0 | 0 | 0 | 0 | 0 | 0 | 0 | 0 | 0 | 0 |
| β-Citronellal | 1141 | 0 | 0 | 0 | 0.06 | 0 | 0 | 0 | 0 | 0.04 | 0.04 | 0.05 |
| (-)-Terpinen-4-ol | 1164 | 1.09 | 0.3 | 0.42 | 0.56 | 0.33 | 0.57 | 0.58 | 0.72 | 0.54 | 0.36 | 0.59 |
| α-Terpieol | 1176 | 0.51 | 0.22 | 0.13 | 0.29 | 0.16 | 0.21 | 0.38 | 0.44 | 0.19 | 0.17 | 0.27 |
| n-Decanal | 1191 | 0 | 0 | 0 | 0.06 | 0 | 0 | 0 | 0.13 | 0 | 0.03 | 0 |
| cis-Carveol | 1202 | 0.44 | 0 | 0 | 0 | 0 | 0 | 0 | 0 | 0 | 0 | 0 |
| β-Citronellol | 1210 | 0.08 | 0 | 0 | 0 | 0 | 0 | 0 | 0 | 0 | 0.01 | 0 |
| β-Citral | 1224 | 0.28 | 0.11 | 0.18 | 0.19 | 0.11 | 0.12 | 0.15 | 0.16 | 0.21 | 0.15 | 0.15 |
| trans-Geraniol | 1236 | 0 | 0 | 0 | 0 | 0 | 0 | 0 | 0 | 0 | 0.2 | 0 |
| α-Citral | 1251 | 0 | 0.12 | 0.23 | 0.22 | 0.11 | 0.16 | 0.17 | 0.2 | 0.24 | 0 | 0.18 |
| p-Cymen-2-ol | 1269 | 0.33 | 0 | 0 | 0 | 0 | 0 | 0 | 0 | 0 | 0 | 0 |
| Nerol acetate | 1334 | 0 | 0 | 0 | 0 | 0 | 0 | 0 | 0 | 0 | 0 | 0 |
| β-Elemene | 1362 | 0.34 | 0 | 0 | 0 | 0 | 0 | 0 | 0 | 0 | 0 | 0 |
| Caryophyllene | 1387 | 0 | 0 | 0 | 0 | 0 | 0 | 0 | 0 | 0 | 0 | 0 |
| β-Farnesene | 1418 | 0.11 | 0 | 0 | 0 | 0 | 0 | 0 | 0 | 0 | 0 | 0 |
| Germacrene D | 1442 | 0.05 | 0 | 0 | 0 | 0 | 0 | 0 | 0 | 0 | 0 | 0 |
| 4-isopropylidene-1-vinyl-o-Menth-8-ene | 1452 | 0 | 0 | 0 | 0 | 0 | 0 | 0 | 0 | 0 | 0 | 0 |
| (Z,E)-α-Farnesene | 1462 | 0.1 | 0 | 0 | 0 | 0 | 0 | 0 | 0 | 0 | 0 | 0 |
| γ-Elemene | 1493 | 0.07 | 0 | 0 | 0 | 0 | 0 | 0 | 0 | 0 | 0 | 0 |
| α-Sinensal | 1567 | 0.04 | 0.04 | 0.24 | 0.08 | 0.11 | 0.07 | 0.21 | 0.06 | 0.02 | 0.02 | 0.05 |

Continues:

| Name | RI | CS12 | CS13 | CS16 | CS17 | CS18 | CS19 | CS20 | CS21 | CS22 | CS23 |
| --- | --- | --- | --- | --- | --- | --- | --- | --- | --- | --- | --- |
| α-Thujene | 943 | 0 | 0.04 | 0.07 | 0.04 | 0.02 | 0.04 | 0.09 | 0.05 | 0.09 | 0.03 |
| α-Pinene | 948 | 0.39 | 0.53 | 0.6 | 0.64 | 0.25 | 0.44 | 0.68 | 0.48 | 0.75 | 0.44 |
| (+)-Sabinene | 979 | 4.83 | 7.71 | 10.35 | 5.86 | 5.3 | 7.52 | 12.42 | 12.82 | 10.88 | 12.26 |
| β-Pinene | 982 | 0.39 | 0.71 | 0.87 | 0.51 | 0.41 | 0.55 | 0.97 | 1.01 | 0.63 | 0.61 |
| β-Myrcene | 992 | 1.11 | 1.31 | 1.29 | 1.21 | 1.08 | 1.25 | 1.51 | 0 | 1.5 | 1.08 |
| n-Octanal | 1002 | 0.14 | 0.07 | 0.11 | 0 | 0.15 | 0.2 | 0 | 0 | 0.06 | 0 |
| α-Phellandrene | 1004 | 0.04 | 0.04 | 0.05 | 0 | 0.04 | 0.03 | 0 | 0 | 0.05 | 0 |
| (+)-4-Carene | 1015 | 0.08 | 0.12 | 0.31 | 0.1 | 0.25 | 0.17 | 0.17 | 0.21 | 0.35 | 0.31 |
| o-Cymene | 1022 | 0 | 0.05 | 0.05 | 0 | 0 | 0 | 0 | 0 | 0.09 | 0.16 |
| Limonene | 1026 | 86.97 | 84.99 | 72.75 | 89.35 | 83.27 | 85.03 | 75.21 | 75.92 | 77.25 | 73.44 |
| β-trans-Ocimene | 1033 | 0 | 0 | 0 | 0 | 0 | 0 | 0 | 0 | 0.62 | 1.09 |
| β-cis-Ocimene | 1042 | 0.53 | 0.27 | 1.02 | 0.13 | 0 | 0.4 | 0.41 | 0.7 | 0 | 0 |
| γ-Terpinen | 1052 | 0.76 | 0.66 | 0.91 | 0.31 | 0.74 | 0.63 | 1.77 | 0.92 | 1.31 | 2.13 |
| cis-β-Terpineol | 1060 | 0.15 | 0.08 | 0.27 | 0 | 0.1 | 0.09 | 0.29 | 0.22 | 0.09 | 0.07 |
| 1-Octanol | 1064 | 0 | 0 | 0 | 0 | 0 | 0.04 | 0 | 0 | 0 | 0 |
| α-Methyl-α-[4-methyl-3-pentenyl]oxiranemethanol | 1065 | 0 | 0 | 0 | 0 | 0 | 0 | 0 | 0 | 0 | 0 |
| p-Mentha-1,4(8)-diene | 1081 | 0.06 | 0.06 | 0.13 | 0.05 | 0.13 | 0.07 | 0.13 | 0.13 | 0.13 | 0.12 |
| β-Linalool | 1089 | 3.57 | 2.29 | 9.21 | 1.34 | 5.14 | 2.29 | 4.72 | 4.67 | 2.72 | 3.25 |
| trans-1-methyl-4-(1-methylethyl)-2-Cyclohexen-1-ol | 1112 | 0 | 0 | 0.05 | 0 | 0 | 0 | 0 | 0 | 0.02 | 0 |
| cis-1-methyl-4-(1-methylethyl)-2-Cyclohexen-1-ol | 1129 | 0 | 0 | 0 | 0 | 0 | 0 | 0 | 0 | 0 | 0 |
| β-Citronellal | 1141 | 0 | 0.05 | 0 | 0 | 0.04 | 0 | 0 | 0 | 0 | 0 |
| (-)-Terpinen-4-ol | 1164 | 0.36 | 0.38 | 1.15 | 0.33 | 1.19 | 0.66 | 0.72 | 0.84 | 1.46 | 2.17 |
| α-Terpieol | 1176 | 0.3 | 0.17 | 0.62 | 0.08 | 0.6 | 0.4 | 0.48 | 0.33 | 0.17 | 0.21 |
| n-Decanal | 1191 | 0 | 0 | 0 | 0 | 0.08 | 0.1 | 0 | 0 | 0.05 | 0 |
| cis-Carveol | 1202 | 0 | 0 | 0 | 0 | 0 | 0 | 0 | 0 | 0 | 0 |
| β-Citronellol | 1210 | 0 | 0 | 0 | 0 | 0 | 0 | 0 | 0 | 0.03 | 0 |
| β-Citral | 1224 | 0.12 | 0.15 | 0.05 | 0 | 0.19 | 0 | 0.15 | 0.08 | 0 | 0 |
| trans-Geraniol | 1236 | 0.13 | 0 | 0 | 0 | 0 | 0 | 0 | 0 | 0 | 0 |
| α-Citral | 1251 | 0 | 0.19 | 0.05 | 0 | 0.21 | 0.09 | 0.17 | 0 | 0.06 | 0.05 |
| p-Cymen-2-ol | 1269 | 0 | 0 | 0 | 0 | 0 | 0 | 0 | 0 | 0 | 0 |
| Nerol acetate | 1334 | 0 | 0 | 0 | 0 | 0 | 0 | 0 | 0 | 0 | 0 |
| β-Elemene | 1362 | 0 | 0 | 0 | 0 | 0 | 0 | 0 | 0 | 0 | 0 |
| Caryophyllene | 1387 | 0 | 0 | 0 | 0 | 0 | 0 | 0 | 0 | 0 | 0 |
| β-Farnesene | 1418 | 0 | 0 | 0 | 0 | 0 | 0 | 0.11 | 0.14 | 0 | 0 |
| Germacrene D | 1442 | 0 | 0 | 0 | 0 | 0 | 0 | 0 | 0 | 0 | 0 |
| 4-isopropylidene-1-vinyl-o-Menth-8-ene | 1452 | 0 | 0 | 0 | 0 | 0 | 0 | 0 | 0 | 0 | 0 |
| (Z,E)-α-Farnesene | 1462 | 0 | 0.03 | 0 | 0 | 0 | 0 | 0 | 0 | 0 | 0 |
| γ-Elemene | 1493 | 0 | 0 | 0 | 0 | 0 | 0 | 0 | 0 | 0 | 0 |
| α-Sinensal | 1567 | 0.14 | 0.2 | 0.04 | 0.02 | 0.03 | 0.01 | 0.02 | 0.25 | 0.04 | 0.05 |

Table 5 PCA loadings plot scores of all the GC-MS signals of the three species of Zhishi samples: 15 samples from *CJ*, 21 samples from *CJ* and 4 samples from *PT*.

| Obs | No. | Lable | Prin1 | Prin2 |
| --- | --- | --- | --- | --- |
| 1 | V17 | p-Mentha-1,4(8)-diene | 0.28503 | -0.09619 |
| 2 | V10 | Limonene | -0.28445 | -0.07717 |
| 3 | V8 | (+)-4-Carene | 0.28233 | -0.11961 |
| 4 | V4 | β-Pinene | 0.28222 | 0.01962 |
| 5 | V13 | γ-Terpinene | 0.27963 | -0.10279 |
| 6 | V1 | α-Thujene | 0.26947 | -0.10831 |
| 7 | V30 | p-Cymen-2-ol | 0.26257 | -0.09057 |
| 8 | V2 | α-Pinene | 0.26193 | -0.10123 |
| 9 | V3 | (+)-Sabinene | -0.23736 | -0.07563 |
| 10 | V9 | o-Cymene | 0.23504 | -0.06822 |
| 11 | V27 | β-Citral | -0.23318 | -0.09272 |
| 12 | V16 | α-Methyl-α-[4-methyl-3-pentenyl]oxiranemethanol | 0.21213 | -0.07286 |
| 13 | V29 | α-Citral | -0.20907 | -0.08049 |
| 14 | V23 | α-Terpieol | 0.20418 | -0.06322 |
| 15 | V39 | α-Sinensal | -0.18495 | -0.05658 |
| 16 | V14 | cis-β-Terpineol | -0.13805 | -0.12484 |
| 17 | V21 | β-Citronellal | -0.09247 | -0.06930 |
| 18 | V24 | n-Decanal | -0.06543 | -0.-6130 |
| 19 | V28 | trans-Geraniol | -0.06254 | -0.02206 |
| 20 | V22 | (-)-Terpinen-4-ol | -0.05652 | -0.03326 |
| 21 | V25 | cis-Carveol | -0.03241 | -0.02116 |
| 22 | V19 | trans-1-methyl-4-(1-methylethyl)-2-Cyclohexen-1-ol | -0.03120 | 0.00074 |
| 23 | V20 | cis-1-methyl-4-(1-methylethyl)-2-Cyclohexen-1-ol | 0.00000 | 0.00000 |
| 24 | V31 | Nerol acetate | 0.00000 | 0.00000 |
| 25 | V35 | Germacrene D | 0.06988 | 0.33981 |
| 26 | V33 | Caryophyllene | 0.02415 | 0.33431 |
| 27 | V12 | β-cis-Ocimene | 0.02355 | 0.33047 |
| 28 | V5 | β-Myrcene | 0.00649 | 0.32416 |
| 29 | V32 | β-Elemene | 0.02332 | 0.30592 |
| 30 | V36 | 4-isopropylidene-1-vinyl-o-Menth-8-ene | 0.03832 | 0.28658 |
| 31 | V37 | (Z,E)-α-Farnesene | 0.02407 | 0.26867 |
| 32 | V7 | α-Phellandrene | 0.01717 | 0.22806 |
| 33 | V38 | γ-Elemene | -0.00004 | 0.21269 |
| 34 | V18 | β-Linalool | -0.07237 | -0.18462 |
| 35 | V34 | β-Farnesene | -0.01099 | 0.15861 |
| 36 | V6 | n-Octanal | -0.05642 | -0.11956 |
| 37 | V15 | 1-Octanol | -0.02865 | 0.04683 |
| 38 | V26 | β-Citronellal | -0.00309 | -0.04000 |
| 39 | V11 | β-trans-Ocimene | -0.01543 | -0.03479 |

1. ^*^ *Corresponding author*:

   Prof. Aiping Lu, Institute of Basic Research in Clinical Medicine, China Academy of Chinese Medical Sciences, Beijing 100700, China. Tel.: +86 10 64067611, Fax: +86 10 64013896.

   *E-mail address*: [lap64067611@126.com](mailto:catcm@public.bta.net.cn) (A.P. Lu).

   ^§^ These authors contributed equally to this work. [↑](#footnote-ref-2)
